# Does laparoscopic Nissen fundoplication prevent the progression of Barrett’s oesophagus? Is the length of Barrett’s a factor?

**DOI:** 10.4103/0972-9941.15242

**Published:** 2005-03

**Authors:** Fahad Bamehriz, Sanjeev Dutta, Catherine Gill Pottruff, Christopher J. Allen, Mehran Anvari

**Affiliations:** Centre for Minimal Access Surgery, McMaster University, Hamilton, Ontario, Canada

**Keywords:** Laparoscopy, fundoplication, gastro-oesophageal reflux disease, Barrett’s oesophagus

## Abstract

**Introduction:**

Recent studies have suggested that both laparoscopic and open anti-reflux surgery may produce regression of Barrett’s mucosa.

**Material and methods;:**

We reviewed 21 patients (13M: 8F, mean age 46.7±3.18 years) with documented Gastroesophageal Reflux Disease (GERD) and Non-dysplastic Barrett’s esophagus (15 patients ?3 cm segment, 6 patients < 3 cm segment) on long term proton pump inhibitor therapy who underwent laparoscopic Nissen fundoplication (LNF) between 1993 and 2000. All patients had undergone pre and yearly postoperative upper GI endoscopy with 4 quadrant biopsies every 2 cm. All patients also underwent pre- and 6 months postoperative 24-hr pH study, esophageal manometry, SF36, and GERD symptom score. The mean duration of GERD symptoms was 8.4±1.54 years pre-operative. The mean follow-up after surgery was 39±6.32 months.

**Results:**

Postoperatively, there was significant improvement in reflux symptom score (37.5 ± 3.98 points versus 8.7 ± 2.46 points, P = 0.0001), % acid reflux in 24 hr (26.5 ± 3.91% versus 2.1 ± 0.84%, P< 0.0001) and an increase in lower esophageal sphincter pressure (3.71 ± 1.08 mmHg versus 12.29 ± 1.34 mmHg, P = 0.0053). Complete or partial regression of Barrett’s mucosa occurred in 9 patients. All patients with complete regression had <4 cm segment of Barrett’s. Progression or cancer transformation was not observed in any of the patients.

**Conclusion:**

LNF in patients with Barrett’s oesophagus results in significant control of GERD symptoms. LNF can prevent progression of Barrett’s oesophagus and in patients with Barrett’s <4 cm may lead to complete regression.

## INTRODUCTION

Gastro-Esophageal Reflux Disease (GERD) is one of the most common chronic disorders of the gastrointestinal tract. An estimated thirty per cent of the general population experience heartburn or acid regurgitation at least once a month.[1] Approximately 10-20% of these patients present with complications of GERD which include esophageal ulceration, stricture, and intestinal metaplasia (Barrett’s oesophagus).[2,3]

Barrett’s oesophagus is recognized as a strong risk factor for esophageal adenocarcinoma,[4] a malignancy that has nearly quadrupled in frequency during the past 2-3 decades.[4,5] The true prevalence of Barrett’s oesophagus is unknown; any estimate depends on the definition of Barrett’s that is used. Its prevalence in normal population is estimated to be 4 per 1000, and up to 20% in patients with chronic GERD.[2,6] It has been suggested that for every known patient with Barrett’s, there might be 20 more unrecognized ones in the general population.[2,7]

While modern medical and surgical anti-reflux therapies are highly effective in controlling GERD symptoms, there is scant evidence that any treatment prevents progression of Barrett’s to dysplasia or adenocarcinoma. In fact, a recent study suggested that medical acid suppression may be associated with increased risk of cancer in patients with severe GERD.[8] This has been attributed to continued bile reflux, a major factor in development of esophageal intestinal metaplasia. Anti-reflux surgery, which controls both acid and bile reflux, may on the other hand prevent progression and cause regression of Barrett’s mucosa.[9,10] DeMeester and colleagues have shown complete loss of intestinal metaplasia in 73% of the patients when the intestinal metaplasia was limited to the cardia compared with 4.4% of the patients with visible Barrett’s oesophagus after laparoscopic and open anti-reflux surgery.[11] Recently, Bowers and his colleagues reported complete loss of intestinal metaplasia in 47% of the patients with Barrett’s oesophagus after laparoscopic anti-reflux surgery.[12] Parrilla *et al* reported that open Nissen fundoplication caused low-grade dysplasia to disappeared in all patients who had low-grade dysplasia Barrett’s oesophagus.[13]

The aim of this study was to review the experience at our institution with non-dysplastic Barrett’s oesophagus in patients treated with laparoscopic Nissen fundoplication, and examines the relative influence of length of Barrett’s mucosa in changes after surgery.

## MATERIAL AND METHODS

### Study population

Twenty-one consecutive patients with documented GERD and non-dysplastic Barrett’s oesophagus who underwent Laparoscopic Nissen Fundoplication (LNF) between 1993 and 2000 were reviewed. A diagnosis of GERD was based on endoscopy, 24hr pH study and manometry findings. Barrett’s oesophagus was diagnosed based on a histopathologic finding of specialized intestinal metaplasia (IM) in endoscopic biopsies. The extent of Barrett’s oesophagus was defined as the distance from the gastro-esophageal junction (the point where the tubular oesophagus joined the most proximal gastric rugal folds) to the location of the highest point of the squamocolumnar junction (the site where the pale white esophageal mucosa met the pink columnar mucosa).

### Symptom score evaluation

Symptom score evaluation was carried out by an independent observer on all patients preoperatively, and 6 months postoperatively using a validated symptom score.[14] Six specific symptoms of GERD, namely heartburn, regurgitation, epigastric or chest pain, epigastric fullness, dysphagia and cough (postprandial or supine), were scored as a product of severity (0-3) and frequency (0-4).

### Quality of life questionnaire

We assessed quality of life using the SF-36 questionnaire. This is a general quality of life instrument with 4 domains of physical health and 4 of mental health. These 8 domains can be summarized into a Physical Health Component score (PCS) and Mental Health Component score (MCS). We used the published scoring algorithms and validated them with test data sets from the publishers of the questionnaire.[15]

### Esophageal manometry and 24-hr pH study

Esophageal manometry was performed with a seven-lumen sleeve-sidehole catheter (Dent Sleeve, Adelaide, Australia). After topical anaesthesia of the nostril, the catheter was introduced nasally and positioned with the sleeve straddling the LES; the patient was then allowed to accommodate to the tube for 20 min. The basal LES pressure was measured by the sleeve sensor, in relation to the gastric pressure. Pressures were measured in the supine position. Drugs that might affect esophageal motility were discontinued for 24h before the study. Manometry was performed after 4-6 h fast and, in smokers, after abstinence from smoking for at least 6 h.

Ambulatory Digitrapper (Synectics, Stockholm, Sweden) was used to perform 24-hr pH testing. The pH probe was positioned 5 cm above the LES, as determined by manometry. Gastroesophageal reflux was defined as a drop in esophageal pH below 4, and the percentage reflux in 24 hours was calculated for each patient. All patients were asked to stop antireflux medication for 5 days before 24-hr pH testing.

### Upper endoscopy and histology

All patients underwent preoperative upper GI endoscopy by a single surgeon (MA) with 4 quadrant biopsies every 2 cm starting at the top of the rugal folds. At endoscopy, the gastroesophageal junction was defined as the point at which the tubular oesophagus ended and the gastric rugal folds began. A columnar-lined oesophagus was identified when the squamocolumnar junction (SCJ) or any part of its circumference extended above the gastroesophageal junction. Patients with an irregular squamocolumnar junction had biopsy samples obtained from glandular mucosal tongues extending into the oesophagus.

### Assessment of progression or regression of Barrett’s mucosa

Standard criteria for endoscopic and histologic assessment were applied. Progression was defined as an increase in the length of the endoscopically visible segment of Barrett’s mucosa, and/or the development of dysplastic changes. Regression was defined as absence of the documented intestinal metaplasia on repeated biopsies with decrease in the length of the endoscopically visible segment.

### Surgery

All patients underwent LNF. The technique has been previously described.[16] The procedure was performed through five cannulae (two 10 mm and three 5 mm). If hiatal hernia is present, the content would be reduced and the sac would be mobilized and would be excised. The oesophagus was mobilized to achieve an intra-abdominal esophageal length of 4-5 cm, partially through the hiatus. No esophageal lengthening procedure was needed. The vagi were identified and protected. Minimal dissection was used behind the oesophagus to create a window large enough to accommodate the fundic pull-through. Short gastric vessels were divided only as necessary to allow a loose wrap without placing tension on the spleen. The superior pole of the fundus was pulled behind the oesophagus through the window created, and three interrupted 2-0 silk sutures were tied intracorporeally to fashion a standard Nissen fundoplication over a 42-52Fr bougie. The sutures were positioned 1-1.25 cm apart, creating a 2.0-3.0 cm wrap, with the most superior suture incorporating a bite of the oesophagus.

### Post-operative care

A water-soluble contrast study was performed on post-operative day 1 to check wrap integrity, rule out leakage, and assess esophageal clearance. Patients were then started on fluid diet and, if tolerating, discharged home on the second postoperative day. Instructions were given to slowly change their intake from pureed to normal food over the ensuing 3 weeks. They were allowed to resume full activity on discharge.

### Follow-up investigations

All patients were invited to undergo 24-hr pH testing, esophageal manometry, GERD symptom score and SF-36 quality of life questionnaire 6 months after the operation. Upper GI endoscope with biopsy was done by the same senior author 6 months after operation and at 2 years and 5 years after surgery.

### Statistical analysis

Statistical analysis was performed using Statview 4.5 (SAS Institute Inc, Cary, NC, USA). All values are expressed as mean (± standard error of the mean). Paired values were compared using Student’s t-test and statistical significance was set at the 0.05 level.

## RESULTS

### Indication for surgery

The 21 subjects included 13 males and 8 females with mean age of 46.8±3.18 years.

The mean duration of GERD symptoms prior to surgery was 8.4 ± 1.54 years. The mean esophageal acid exposure was 26.5 ± 3.91%. Upper GI endoscopy showed 15 patients with Barrett’s segment ≥3 cm and 6 patients with Barrett’s segment <3 cm. 18 patients had hiatal hernia with range of 3-8 cm. 7 patients had erosive esophagitis. No esophageal stricture was identified. Indications for surgery in our sample included failure of medical therapy in 10 patients, pulmonary complications of GERD in one patient, and in the remaining 10 patients, surgery was chosen over long term PPI therapy despite good symptom control.

### Effect of surgery on reflux indices

**Figure 1:**
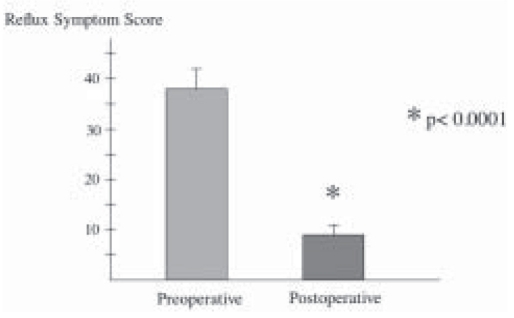
Control of reflux symptoms before and 6 months after antireflux surgery

**Figure 2:**
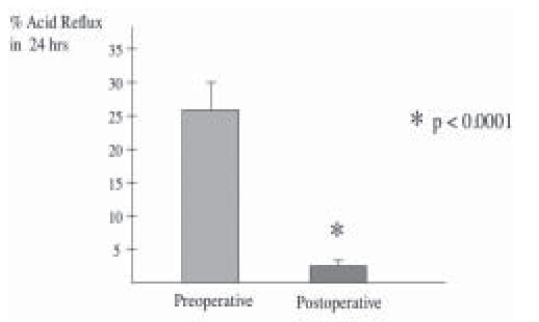
Percent acid reflux in 24 hrs before and 6 months after antireflux surgery

**Figure 3:**
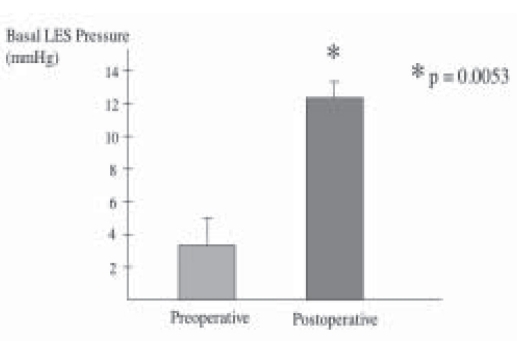
Lower esophageal sphincter pressure before and 6 months after anti-reflux surgery for

All patients underwent Laparoscopic Nissen fundoplication (LNF) by a single surgeon with mean operative time of 63.3 ± 6.14 minutes. There was no conversions and the mean hospital stay was 2.9 ± 2.46 days. Mean follow-up was 39 ± 6.32 months. Reflux symptom score (off antisecretory medications) at 6 months was significantly improved from 37.5 ± 3.98 to a mean of 8.7 ± 2.46, *P* < 0.0001 (Figure 1). This was supported objectively by a significant drop in the esophageal acid exposure time from mean of 26.5 ± 3.9% to a value of 2.1 ± 0.84%, *P* < 0.0001 (Figure 2). Esophageal manometry showed significant augmentation of the lower esophageal sphincter from mean of 3.71 ± 1.08 mmHg to a mean basal pressure of 12.29 ± 1.34 mmHg, *P*<0.0053 (Figure 3). Both the physical and mental components of SF- 36 were improved from means of 44.8 ± 1.70 and 50.17 ± 2.5 respectively to means of 50.32 ± 2.43 and 55.81 ± 1.45 points respectively, *P* <0.0673 and *P* <0.0751 (Figure 4a–4b).

**Figure 4a:**
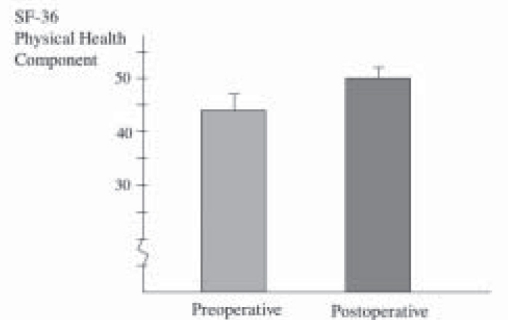
Quality of life-physical component before and 6 months after anti-reflux surgery

**Figure 4b:**
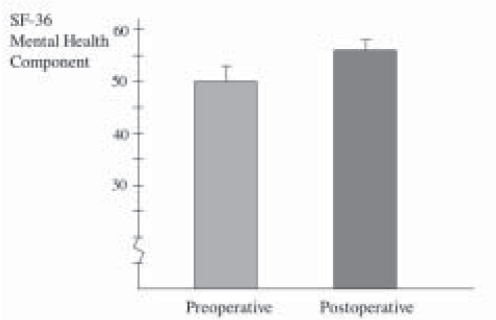
Quality of life-mental component before and 6 months after anti-reflux surgery

### Effect of surgery on Barrett’s mucosa

**Figure 5:**
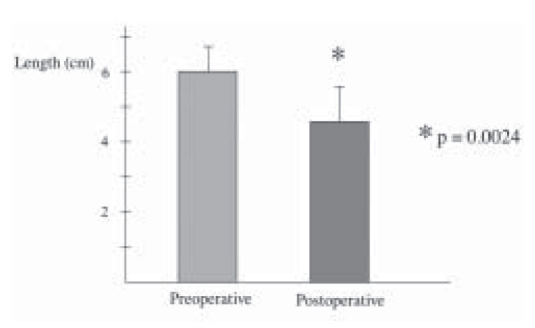
Length of Barrett’s mucosa before and after anti-reflux surgery

Of the 21 patients, none have developed dysplastic changes nor exhibited progress of the length of intestinal metaplasia segment of Barrett’s. In 9 patients we have observed regression of Barrett’s mucosa, of these 8 patients have shown complete loss of documented intestinal metaplasia on the most recent biopsies after a median follow-up of 3 years (Table 1) (Figure 5). These 8 patients included 6 patients with Barrett’s <3 cm and 2 patients with Barrett’s ≤4 cm. The one patient who exhibited partial regression had a reduction in visible length of intestinal metaplasia segment from 12 cm to 5 cm on biopsy after 5 years of follow-up.

**Table 1:** Effect of surgery on progression of Barrett’s

Patients with Barrett’s n=21	Complete regression	Partial regression	No regression	Progression
Barrett’s < 3 cm n=6	6	0	0	0
Barrett’s ≥3 cm n=15	2(both < 4 cm)	1	12	0

## DISCUSSION

The goal of medical and surgical treatment of Barrett’s oesophagus is to provide long-term relief of GERD symptoms, control esophagitis, and hopefully prevent progression of Barrett’s mucosa that may lead to esophageal adenocarcinoma.

Clinical studies have shown that PPI therapy prevents acid reflux and fasting bile reflux but does not prevent postprandial bile reflux,[17] and there is only equivocal evidence that it works to prevent progression of Barrett’s mucosa.[15,18,19] In fact, there is evidence to suggest that acid suppression therapy may contribute to cell proliferation and adenocarcinoma development.[20] This observation has been attributed to the carcinogenic effects of unconjugated bile acids, which are most injurious to the esophageal mucosa at alkaline pH.[21] Acid suppression therapy promotes an alkaline environment for these unconjugated bile acids which can reflux in to the oesophagus. Surgical therapy on the other hand, is effective in preventing both acid and bile reflux,[22] and hence minimizes exposure of the esophageal mucosa to these harmful bile acids. Thus, theoretically it should be more effective than PPI therapy in preventing progression and even causing regression of Barrett’s. There is increasing evidence in favor of this theory.

### Effect of surgical therapy on causing regression of Barrett’s

There is increasing evidence in favor of surgical therapy causing regression of Barrett’s. Pope *et al* were the first to describe a complete regression of intestinal metaplasia in 4 of 10 patients with Barrett’s who underwent successful open antireflux surgery.[23] Low *et al* also reported complete regression of Barrett’s oesophagus in 2 of 14 patients, partial regression in 10 of 14 patients, and disappearance of dysplasia in 4 of 14 patients, who were followed up for a mean of 25 months after open antireflux surgery.[24] DeMeester recently demonstrated that both 73% of patients with intestinal metaplasia at the gastroesophageal junction and 4.4% of patients with an endoscopically visible segment of Barrett’s had complete regression and loss of intestinal metaplasia after laparoscopic antireflux surgery.[11] The only randomized study during open antireflux surgery period was reported by Ortiz and colleagues.[19] They randomized 27 patients to the medical arm with 4 year follow-up and 32 patients to the surgical arm with 5 year follow-up. Regressions were documented in 2 of 27 patients of medical arm and in 8 of 32 patients of surgical arm. Hofstetter *et al* reported the outcome of 97 patients with Barrett’s after antireflux surgery. Fifty patients underwent a laparoscopic antireflux surgery. 85 patients completed median follow-up of 5 years, intestinal metaplasia regressed to cardiac mucosa in 9 of 63 (14%) patients and low-grade dysplasia regressed to nondysplastic Barrett’s in 7 of 16 (44%) patients.[3] Bowers *et al* reported the results of 104 patients with Barrett’s oesophagus, in whom 97 patients underwent laparoscopic antireflux surgery, followed for a mean of 4.6 years. Of the 66 patients who remained on the surveillance protocol, 31 (47%) patients documented loss of intestinal metaplasia in tubular oesophagus.[12] Recently, Parrilla and his colleagues showed that, open Nissen fundoplication in 57 patients who had Barrett’s oesophagus without esophageal stricture, complete disappearance of low-grade dysplasia after successful anti-reflux surgery.[13] Our study confirms the finding of these studies in that laparoscopic Nissen fundoplication resulted in complete loss of intestinal metaplasia in 8 of 21 (38%) patients and partial regression in one patient. This can be attributed to the prevention of esophageal exposure to carcinogenic bile acid. This effect seems to be most pronounced in those patients with segment of Barrett’s ≤4 cm, suggesting that surgical therapy may be considered as a first line approach in patients with a short segment of Barrett’s.

The evidence in support of surgery in reducing the risk of cancer development is even scarcer. McCallum *et al*[9] enrolled 338 patients with Barrett’s, with 256 patients were non-dysplastic Barrett’s on initial entry. 40 patients had open anti-reflux surgery and 216 patients continued on medical therapy. Only 29 patients of the surgical group had mean follow-up of 62 months and none developed cancer. 152 patients of medical group had mean follow-up of 49 months and 2 patients developed esophageal adenocarcinoma. Similarly, Katz *et al*[10] retrospectively followed 102 patients with Barrett’s for a mean of 4.8 years. Dysplasia had developed in approximately 8% of the medically treated patients compare to none of the 15 patients group, whom were treated by antireflux surgery and were matched in this study, developed dysplasia nor cancer. DeMeester *et al*[11] followed 60 patients with intestinal metaplasia of the oesophagus or cardia who had antireflux surgery. 15 patients had only intestinal metaplasia of the cardia and 45 patients had columnar epithelium with intestinal metaplasia visible within the oesophagus. After a median follow-up of 25 months in each group, no patient progressed to high-grade dysplasia or cancer. Hofstetter *et al*[3] followed 85 patients for a median of 5 years. In this study 50 patients underwent a laparoscopic procedure, 20 had a transthoracic procedure, 3 had abdominal Nissen operations, 9 had Collis-Belsey, and 3 had other partial wrap. No patient developed high-grade dysplasia or cancer in 410 patient-years of follow-up. Bowers *et al* followed 104 patients for a mean of 4.6 years. In this study 96 patients underwent a laparoscopic antireflux procedure. There was no progression of intestinal metaplasia to adenocarcinoma or high-grade dysplasia in any of the patients, for a total of 337 patients-years of follow-up.[12] Krska *et al* prospectively followed 75 patients with GERD. 8 patients had Barrett’s oesophagus and underwent LNF. They did not observe any one case of Barrett’s segment progression.[25]

None of our patients developed dysplasia or cancer during a mean follow-up of more than 3 years. This finding supports the previous reports that high-grade dysplasia and adenocarcinoma can be prevented. However, as with all other studies, our series is relatively small and the folloup is just over three years. A larger study, preferably a randomized controlled trial with a medical arm over a long followup period, may help confirm the recent evidence in favour of surgery in the treatment of Barrett’s.

There are few studies that document the symptomatic outcome in patients with Barrett’s oesophagus after antireflux surgery. McDonald *et al* retrospectively reviewed 113 patients with Barrett’s oesophagus underwent open antireflux surgery. 82.2% of the patients had excellent to good control of symptomatic outcome during median follow-up of 6.5 years after the surgery.[26] Parrilla *et al* divided 177 patients into two groups: 57 patients had Barrett’s oesophagus (BE) and 120 patients had no Barrett’s oesophagus. Both groups underwent open antireflux surgery. 92% of patients with BE had excellent to good clinical response during mean follow-up of 5 years after surgery compare to 90% of patients without BE who had same response during mean follow-up of 6 years after surgery.[13] Farrell and his colleagues compared symptom scores and reoperation rates in 570 patients who had GERD (74 patients had Barrett’s oesophagus and 496 controls). All patients underwent fundoplication. During mean follow-up of 4 years after the surgery, the heartburn, regurgitation, and dysphagia severity scores of the Barrett’s group was significantly improved and was identical to the controls.[27] The report by Williamson *et al*[28] of 37 patients with median follow-up of 5 years showed symptomatic relief of esophagitis in 92% of patients. Yau *et al*[29] published the outcome of a prospective series of 81 patients who had Barrett’s oesophagus undergoing laparoscopic antireflux surgery compared with 676 patients who did not have Barrett’s oesophagus. After a median follow-up of 2 years, 6 (7%) of the patients underwent a subsequent operations and the outcomes in regard to symptom score, dysphagia score, and satisfaction index were all favorable, with no statistical significance compared with individuals who did not have Barrett’s oesophagus. In the study done by Hofstetter *et al*,[15] reflux symptoms were absent in 67 of 85 (79%) patients at a median follow-up of 5 years after the surgery. Bowers *et al* followed a total of 104 patients with Barrett’s oesophagus who underwent fundoplication (97 patients underwent laparoscopic anti-reflux surgery). During mean follow-up of 4.6 years, only 27% of the patients reported moderate to severe reflux symptoms after the surgery.[12]

We documented a significant improvement of reflux symptom score after surgery which supports earlier findings that the result of laparoscopic antireflux surgery in patients with Barrett’s is favourable. We will continue to follow the patients as more long term follow up will be necessary.

Assessing quality of life in GERD patients with Barrett’s following fundoplication is rarely reported or studied. Very few studies evaluated this point. Bowers *et al* reported the clinical results of 104 patients with Barrett’s oesophagus who underwent fundoplication. At mean follow-up of 4.6 years after surgery, 45 patients reported statistically improvement in seven of eight quality of life fields of SF-36 questionnaire. Comparing them to age-matched controls from the general population, 45 patients group were statistically identical to the match group.[12] Kamolz *et al*[30] prospectively studied 249 patients (75 patients had Barrett’s oesophagus and 174 patients had no Barrett’s) who underwent laparoscopic anti-reflux surgery. He evaluated quality of life, using Gastrointestinal Quality of Life Index (GIQLI), for both groups (BE vs Non BE) preoperatively, 3months, 1 year, and 3 years after surgery and compared the data with general population. GIOLI was significant improved in both group after surgery and there were no differences in comparing with general population. He reported that laparoscopic anti-reflux surgery significantly improve the quality of life in GERD patients with Barrett’s oesophagus. In our study, both the physical and mental components of SF-36 were improved after LNF which is supporting the previous reports.

There are only a few studies which have evaluated the possible impact of length of Barrett’s on its regression after surgery. DeMeester *et al*[11] reported complete loss of intestinal metaplasia in 11 of 15 (73%) patients with intestinal metaplasia of the cardia after antireflux surgery. Complete regression of intestinal metaplasia were also occurred in 2 of 45 (4.4%) patients with Barrett’s but both patients had columnar segments less than 3 cm in length. No patient with a segment of columnar epithelium 3 cm or longer had complete loss of intestinal metaplasia. Low *et al*[24] reported a complete regression of intestinal metaplasia in 2 of 14 (14%) patients with short-segment Barrett’s oesophagus (less than 3 cm) after antireflux surgery. 4 of 14 (28%) patients regressed from low-grade dysplasia to nondysplastic Barrett’s. In the study done by Bowers *et al*, 31 patients with loss of intestinal metaplasia had mean length of preoperative visible columnar-lined epithelium of 2.8 ± 2.4 cm, in comparison to 33 patients with persistent intestinal metaplasia who had mean length of preoperative visible columnar-lined epithelium of 5.5 ± 3.7 cm, (*P* value was < 0.01). He reported that patients with short-segment (<3 cm) Barrett’s oesophagus were more likely to have regression of Barrett’s segment after antireflux surgery than those with long-segment (>3 cm) disease.[12]

Our study confirms only patients with relatively short segments of Barrett’s (<4 cm) may experience complete regression. This observation suggests that the metaplastic process may indeed be reversible if reflux-induced injury is eliminated early in its process.

More studies are required to evaluate this hypothesis and determine the impact of laparoscopic antireflux surgery on long-term survival in patients with Barrett’s oesophagus.
